# Bioimaging of Dissolvable Microneedle Arrays: Challenges and Opportunities

**DOI:** 10.34133/2022/9758491

**Published:** 2022-08-01

**Authors:** Yanni Wang, Gehua Ma, Guangzhi Gao, Ji Tao, Wenzhao Cao, Haohao Sun, Fengsen Ma, Yilong Zhang, Yen Wei, Mei Tian

**Affiliations:** ^1^Laboratory of Biologics and Biomaterials, College of Pharmacy, Zhejiang University of Technology, Hangzhou 310014, China; ^2^College of Computer Science and Technology, Zhejiang University, Hangzhou 310027, China; ^3^Human Phenome Institute, Fudan University, Shanghai 201203, China; ^4^College of Information Engineering, Zhejiang University of Technology, Hangzhou 310023, China; ^5^Life Science Research Center, Frontier Crossing Institute, Zhejiang University of Technology, Hangzhou 310023, China; ^6^Engineering Research Center of Intelligent Sensing and System, Ministry of Education, Hangzhou 310023, China; ^7^College of Computer Science and Technology, Zhejiang University of Technology, Hangzhou 310023, China; ^8^Department of Chemistry and the Tsinghua Center for Frontier Polymer Research, Tsinghua University, Beijing 100084, China

## Abstract

The emergence of microneedle arrays (MNAs) as a novel, simple, and minimally invasive administration approach largely addresses the challenges of traditional drug delivery. In particular, the dissolvable MNAs act as a promising, multifarious, and well-controlled platform for micro-nanotransport in medical research and cosmetic formulation applications. The effective delivery mostly depends on the behavior of the MNAs penetrated into the body, and accurate assessment is urgently needed. Advanced imaging technologies offer high sensitivity and resolution visualization of cross-scale, multidimensional, and multiparameter information, which can be used as an important aid for the evaluation and development of new MNAs. The combination of MNA technology and imaging can generate considerable new knowledge in a cost-effective manner with regards to the pharmacokinetics and bioavailability of active substances for the treatment of various diseases. In addition, noninvasive imaging techniques allow rapid, receptive assessment of transdermal penetration and drug deposition in various tissues, which could greatly facilitate the translation of experimental MNAs into clinical application. Relying on the recent promising development of bioimaging, this review is aimed at summarizing the current status, challenges, and future perspective on in vivo assessment of MNA drug delivery by various imaging technologies.

## 1. Introduction

**Figure 1:**
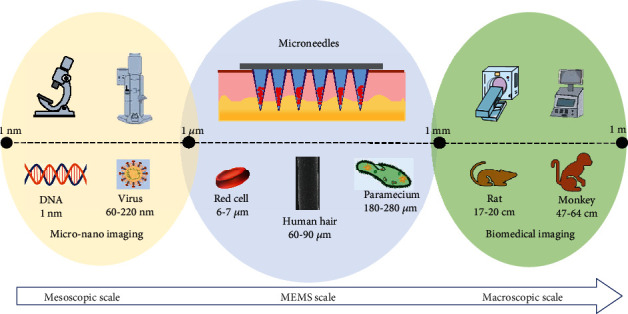
Schematic diagram of MNA imaging analysis in the size dimension between macroscopic and mesoscopic scales.

Microneedle arrays (MNAs) are emerging as a promising physical enhancement technique for transdermal delivery systems, which is capable for breaching the skin barrier to controlled bioactive ingredients release and delivery. Compared with the conventional injections, MNA-based drug delivery has the advantages such as reduction of pain, minimal invasiveness, high efficiency, better patient compliance, and intelligent response [1, 2]. Typically, a patch of MNAs consists of tens to hundreds of needles 300-1000 *μ*m long with 1-20 *μ*m tips attached to a backing layer. The size of microneedles lies between the macroscopic (0.1 mm-1 km) and mesoscopic (1-100 nm) scales of matter, similar to that of microelectromechanical systems (MEMS), which is between submicron (0.1 *μ*m-1 *μ*m) to submillimeter (0.1 mm-1 mm) (Figure 1). Thus, the evaluation of micron-sized MNA drug delivery system requires high spatial resolution of transdermal penetration depth and high sensitivity of imaging agent, which differs significantly from those used for conventional drug delivery systems in animals and humans [3]. The observation of molecular or cellular microstructures from nanometer to a few microns in size usually requires the assistance of electron microscope (EM), or super-resolution microscopy (SRM) which is beyond the diffraction barrier and enhances the spatial resolution [4]. In contrast, macroscopic optical imaging focuses on the whole body. Other imaging modalities including digital radiography (DR), computed tomography (CT), magnetic resonance imaging (MRI), positron emission computed tomography (PET), and PET-CT have been extensively applied in clinical practice with favorable tissue penetration depth and limited spatial resolution at the millimeter level [5]. The depth of penetration, signal-to-background contrast, and spatial resolution are essential for a promising imaging technique. Evaluating the tissue penetration performance of micro-nanoscale medical products like MNAs particularly requires the ability to balance imaging depth and resolution, which is a challenge for imaging technology. These demanding requirements are equally important for studying the in vivo kinetics of other MEMS-scale products formed by bioactive components in tiny spaces, such as biosensors, biochips, smart wearable devices, and intelligent drug delivery systems [6–10]. Advanced imaging techniques are essential to identify and characterize these multiscale and multilevel macroscopic or microscopic changes [11].

**Figure 2:**
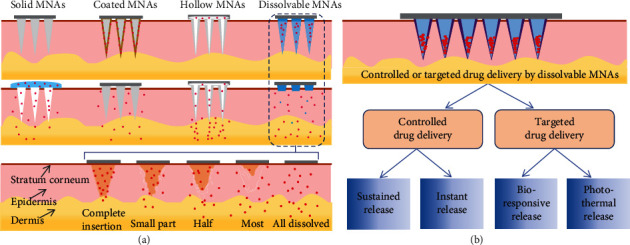
MNAs applied to transdermal delivery of bioactive substances. (a) Schematic diagram of the different types of MNAs: solid (made of metal, monocrystalline silicon, or insoluble material), coated (drug adsorbed on solid MNAs, also the hydrogel-formed MNAs), hollow (microneedles for injection, which have to be removed after insertion) and dissolvable polymeric MNAs, whose dissolution process after insertion is illustrated. (b) Dissolvable MNAs for controlled release and targeted delivery of drugs.

**Figure 3:**
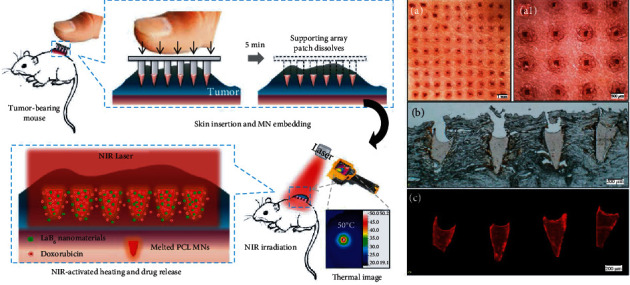
Skin insertion ability of MNAs. (a and a1) Porcine cadaver skin after insertion of DOX-loaded MNAs and their corresponding histological sections (b and c): (a) low magnification; (a1) high magnification. (b) Bright-field images. (c) Fluorescence images [22], reprinted with permission from the American Chemical Society.

Dissolvable MNAs, also known as polymeric MNAs, including those of soluble and biodegradable, are considered a more acceptable option than injections in delivering drugs, vaccines, and cosmetics to the skin, paving a new pathway for improving patient compliance and achieving on-demand drug release [12, 13] (Figure 2). Typically, microneedle can be a homogeneous needle body or loaded with various nanoparticles that, when pierced into the body, involves a series of complex processes such as absorption, swelling, dissolution, degradation, and diffusion to achieve instant, sustained, or targeted release of the drug [13, 14] (Figures 2 and Figure 3). Dissolvable MNAs are mainly composed of polymers including chitosan, hyaluronic acid (HA), cellulose derivatives, and synthetic polymeric materials such as polyvinylpyrrolidone (PVP), polylactic acid-hydroxyacetic acid (PLGA) [12, 14, 15], and it is noteworthy that the mechanical strength is not as strong as that of metal or monocrystalline silicon. The penetration depth of dissolvable MNAs into the skin determines the drug delivery efficiency; thus, further research is needed to make use of optimized imaging methods to better identify the insertion performance of MNAs. The assessment of skin penetration capability of dissolvable MNAs has received much attention in recent years [16–19]. Imaging techniques that can provide millimeter-level depth and micron-level resolution, as well as noninvasive and real-time observation, will play a significant role in the objective evaluation of microneedle penetration effects, pharmacokinetic studies, and the establishment of quality control system for MNAs.

The previous approaches used to evaluate the penetration effect of MNAs were mainly conventional imaging techniques, such as optical microscopy or fluorescence microscopy (for observing the results of parafilm puncture or tissue sections) and ultrasound imaging (USI). Optical coherence tomography (OCT) [20, 21], confocal laser scanning microscopy (CLSM), CT, photoacoustic microscopy (PAM), and two-photon microscopy (TPM) are advanced imaging methods that can provide more detailed imaging results or rely on more sophisticated techniques. The operating frequencies of USI and MRI are MHz-level (millimeters or submillimeter), those of OCT/CLSM/TPM are mainly in the range of hundred nanometers or micrometers, and CT operates at frequencies commonly referred to as ultrashortwave X-rays of less than 10 nm. All the imaging modalities mentioned above differ in their effective detection depth, spatial resolution, accuracy, and real-time.

Understanding the application of imaging techniques and tracking those applicable and advanced real-time imaging techniques, such as OCT, can not only help us improve the objective analysis of dissolvable MNA penetration depth and drug distribution to guide the design, optimization, and refinement of MNA products to better meet clinical needs, but also provide new directions for improvement of general imaging tools from the perspective of this unique application and expand their further practical applications in the fields of medicine and life sciences.

## 2. Conventional Imaging Technologies

**Figure 4:**
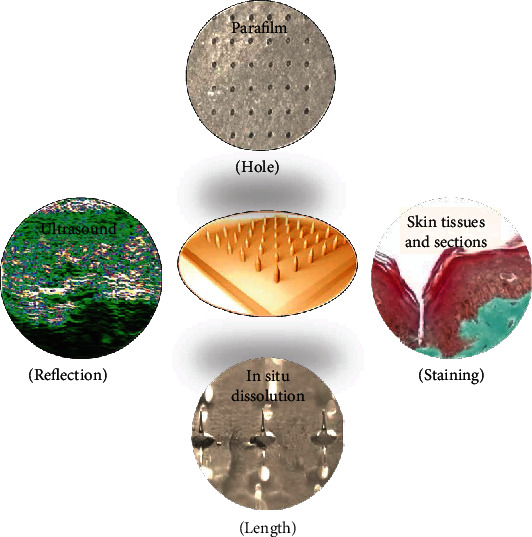
Conventional imaging and related assessment methods for skin puncture evaluation of MNAs including parafilm puncture, histological sectioning method [23], reprinted with permission from John Wiley and Sons; ultrasound imaging [24], reprinted with permission from John Wiley and Sons; and in situ dissolution method [25], reprinted with permission from John Wiley and Sons.

The adoption of optical imaging and ultrasonography etc. are all conventional imaging methods for evaluating MNA insertion depth (Figure 4). Among optical methods, the parafilm puncture is widely used as an in vitro approach to simulate skin puncture, and tissue sectioning is the most classic imaging method.

### 2.1. Optical Imaging

The observation was done after the MNAs penetrate into the parafilm and the skin is mostly carried out with an ordinary optical microscope, and the different magnifications can be obtained (Figure 3 (a and a1)). The parafilm is a flexible thermoplastic sheet made of olefin materials, which is usually used as an artificial skin model to evaluate the penetration depth of MNA puncture in vitro [30]. Generally, eight layers of parafilm were superimposed and subsequently fixed on a polystyrene plastic foam plate or other materials. The MNAs were inserted into the aforementioned layer under a certain external force. Finally, the puncture of each layer was observed with an optical microscope, and the number of layers punctured was counted to estimate the penetration depth (Figure 4). The number of holes in the parafilm was easily visible to the naked eye. For a more convenient observation, the parafilm layer can be placed between two polarizing filters [31], or the Zeta profiler [32] can be applied to identify the number of holes. At the same time, the size of the pores in the parafilm can also be utilized to characterize the uniformity of the needle's diameter and length of the MNAs [33]. Simple and intuitive, easy feasible to operate, and inexpensive are of utmost advantages for such a method. However, when superimposed on a parafilm with a thickness of more than 120 *μ*m, the underlying layer can sometimes only be concave rather than perforated by the MNAs, which inevitably leads to counting errors. Hence, the search for novel and more suitable alternatives is urgently needed [19].

**Figure 5:**
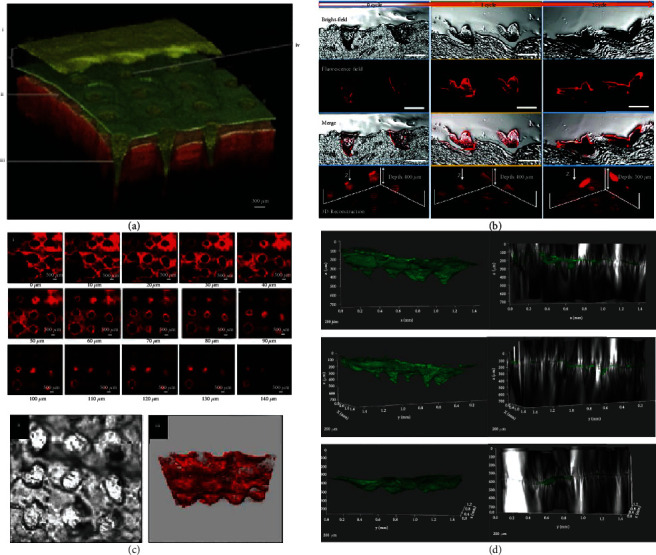
3D reconstruction of multiple imaging techniques after MNA insertion. (a) 3D reconstruction map of OCT images showing MNAs (height 600 *μ*m, width at base 300 *μ*m, and spacing 300 *μ*m) inserted into the human skin in vivo [26], reprinted with permission from Springer Nature. i: MNA base-plate, ii: in situ microneedle channel, iii: stratum corneum, and iv: microneedle-induced hole in stratum corneum. (b) Histological sections of MNA arrowheads inserted into the skin with 0, 1, and 3 cycles of NIR, scale bar: 200 mm. 1st line: bright-field images; 2nd line: fluorescence-field images; 3rd line: merged images; and 4th line: 3D reconstruction images) [27], reprinted with permission from Royal Society of Chemistry. (c) Confocal image of doxorubicin and docetaxel dissolvable microneedles insertion [28], reprinted with permission from Elsevier. i: Confocal micrographs of optical sections of the skin sample from the surface (0 *μ*m) to 140 *μ*m inside of the skin and a representative bright-field image of the pores created with the microneedle insertion. ii: 3D representation of the microchannels created with the insertion of microneedles. iii: Dark areas indicate lack of fluorescence. The scale bar represents 500 *μ*m. (d) 3D visualization of FITC-OVA NP-loaded MNA infiltration in porcine sclera at specific time intervals (1, 6, and 24 h), reconstructed from the multiphoton microscopy images [29], reprinted with permission from Elsevier.

Histological sectioning is a common approach in pathological research, and it is also selected for evaluating the penetration depth of MNAs [34]. The skin punctured by the MNAs is usually cut into 6-12 *μ*m slices, stained with hematoxylin and eosin (H&E) or fluorescent dyes, and then observed under light or fluorescence microscopy as well as a panoramic scanner [22, 35] (Figures 3 and 5(b)). Through the corresponding software processing and analysis, the size of the microchannel and finally the penetration depth can be measured. Park et al. [36] used carboxymethyl cellulose MNAs to penetrate pig skin and the penetration depth was about 200 *μ*m, but the slices obtained here were deformed. Compared with the preceding paraffin puncture method, the histological section provides a more accurate and intuitive evaluation, but the skin sample may be deformed and displaced during the preparation, leading to the results that are at variance with reality. Furthermore, staining is also a significant factor for imaging. More research involving imaging optimization is focused on virtual imaging to obtain higher image quality. Researchers have tried to use conditional generative adversarial networks (cGANs) to accept the autofluorescent of nonstained biological tissue with whole slide images and computationally stain them by learning hierarchical nonlinear mappings between image pairs before and after H&E staining [37]. A recent study presented the ability to create a label-free virtual H&E image, but it requires physical contact between the ultrasonic transducer and the sample to measure the generated sound waves [38]. In general, the slice imaging process based on algorithm analysis faces the time and economic pressures.

### 2.2. Ultrasound Imaging

Ultrasound imaging (USI), based on the detection of objects and measurement of distances by ultrasound, is usually applied for monitoring industrial product quality [39] and clinical disease diagnosis [40] (Figure 4). Indeed, USI was often used as an external auxiliary means to overcome the obstacles of the stratum corneum to promote the permeability of drug transdermal delivery [41]. Nguyen and Banga [33] used the DermaScan ultrasonic diagnostic instrument to perform USI of the isolated skin punctured by the PLGA MNAs for the first time and to evaluate the microporous channels in the skin. However, due to the low-resolution and unclear imaging, the apparatus can only provide a qualitative rather than a quantitative measurement of microchannel size, and it is difficult to clearly distinguish the various layers of the skin.

### 2.3. Magnetic Resonance Imaging

A recent study reported a programmable polymeric MNA made of PVP, polydopamine/manganese dioxide (termed PDA@MnO2), and methotrexate for the treatment of rheumatoid arthritis (RA) [42]. PDA@MnO2 can be used as an MRI contrast agent in the synovial microenvironment of RA. Other researchers performed the depth statistics after the MRI of cross-section of PLGA MNAs inserted into the skin of pigs and obtained the results of penetration depth equivalent to 75% of the total needle length [43]. Also, the dyes such as trypan blue were employed to stain the skin and observed under an ordinary microscope, presenting the overall puncture effect of an MNA patch [33, 44]. However, it is difficult to accurately measure the ratio of the skin portion punctured inside to that remaining outside.

It can be estimated the length penetrated of the microneedles after piercing the skin by subtracting the residual needle body from the total length (Figure 4) [45], and quantification is typically performed using optical microscopy coupled with comprehensive imaging software [46]. Although this method is easy to implement, it is almost impossible to know the exact penetration depth and amount due to unpredictable factors such as the occurrence of mechanical damage during insertion or extraction and the partial dissolution of microneedles on the skin surface, all of which add to the complexity. Thus, the measurement of residual length based on microscopic imaging techniques to study the penetration of MNAs and their length over time is still quite good.

## 3. Advanced Imaging Technologies

The above imaging techniques used for evaluation MNA penetration efficiency are either cumbersome and time-consuming to produce, such as tissue sections, or have low accuracy as in the case of parafilm puncture. USI, although capable of providing real-time observations, does not meet the evaluation requirements due to the low imaging resolution. There is a need to rely on more advanced and effective imaging techniques to address some or all of these challenges. OCT, CLSM, TPM, PAM, and CT are some excellent techniques and hold the crucial advantages of providing real-time, noninvasive, in situ, or in vivo images of the MNA penetration, dissolving, or swelling in the skin.

### 3.1. Two-Photon Fluorescence Imaging

The penetration depth of two-photon microscopy (TPM) imaging varies widely between tissue types, with hundreds of micrometers deep cell imaging in various organs of living animals [47], and for highly transparent tissues (e.g., the cornea), imaging depths of more than 1 mm can be achieved [48]. Current studies of TPM for MNA penetration depth usually rely on the imaging generated by transdermal action of the drug loaded by MNAs [49]. Chiu et al. [50] used TPM to image and observed the microholes punctured by the MNAs. The observation provided further evidence that nanoparticles can function as reservoirs for lipophilic drugs and thereby enable the sustained and controlled delivery of multiple compounds. Notably, TPM imaging should be performed 20-30 minutes after applying MNAs owing to excessive sample preparation time [51]. TPM microscopy can be well imaged with the second harmonic of the skin tissue itself, which makes the collagen fibers in the dermis fluoresce blue, while the stratum corneum has strong green autofluorescence and the epidermis has no fluorescent signal, so that the various tissue layers of the skin show different color signals, which has obvious advantages compared to fluorescent dye-stained imaging specimens.

### 3.2. Photoacoustic Imaging

Photoacoustic imaging (PAI) techniques such as photoacoustic tomography (PAT), photoacoustic computed tomography (PACT), and photoacoustic microscopy (PAM) have the unique ability to penetrate deeper and sustain higher spatial resolution. Compared to USI, PAT has rich intrinsic and extrinsic optical contrasts and is free of speckle artifacts that occur during OCT translucent tissue imaging [52]. The penetration depth is less than 100 *μ*m in acoustic resolution photoacoustic microscopy (AR-PAM), while a lateral resolution up to 0.5 *μ*m and a maximum of 1.2 mm inside the biological tissue [53] can be achieved in optical resolution photoacoustic microscopy (OR-PAM).

**Figure 6:**
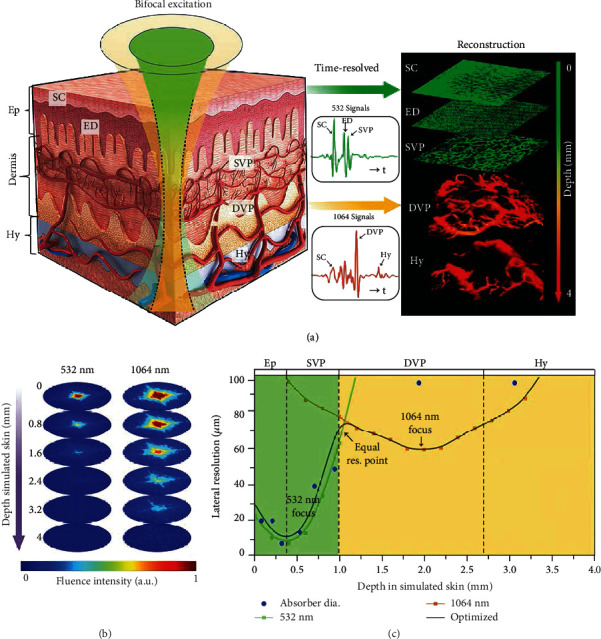
Bifocal photoacoustic microscopy system using two excitation beams. (a) The schematic for the dual-wavelength laser excitation and photoacoustic image reconstruction of BF-PAM. (b) The simulated transmission optical intensity map of 532 nm laser and 1064 nm laser beams at different depths in the simulated skin. (c) The lateral resolution varies with the depth in the simulated skin and the diameters of main absorbers of the human skin in different skin layers. Ep: epidermis; Hy: hypodermis; SC: stratum corneum; SB: stratum basale; ED: epidermal-dermal junction layer; SVP: superficial vascular plexus; DVP: deep vascular plexus [54], reprinted with permission from John Wiley and Sons.

In vivo structural, functional, molecular, and cellular imaging has made extensive use of PAI [54–56] (Figure 6). PAI has been successfully applied to characterize the transdermal delivery of nanoparticles using MNAs [57]. Additionally, structured illumination microscopy (SIM) has enabled wide-field fluorescence imaging to break through the resolution limitations of conventional lenses and has become a key technique for the observation of microscopic objects in cell biology. Ferrara et al. [58] used the optical principle of SIM to facilitate the resolution of USI by manipulating the transmitted acoustic field and mixing high spatial frequency codes into the image. SIM also has been shown to be able to provide accurate lifetime measurements when used in conjunction with fluorescence lifetime imaging microscopy (FLIM). The intensity of a fluorophore depends on its concentration. However, the lifetime of a fluorophore is mostly independent of its concentration. The fluorescence lifetime is also independent of the irradiated laser intensity and photobleaching, which makes fluorescence lifetime imaging advantageous in thick tissue imaging [59].

### 3.3. Confocal Laser Scanning Microscopy

Confocal laser scanning microscopy (CLSM) achieves optical sectioning of thick specimens by eliminating the defocus signal and can realize imaging scanning for both ex vivo and in vivo skin. Moreover, CLSM can provide images of the epidermal and upper dermis cell layers and distinguish the blood flow in the capillaries in each dermal papilla and identify individual circulating blood cells with high resolution. Imaging at a depth of about 100-350 *μ*m below the stratum corneum allows the detection of collagen fibers, sebaceous glands, sweat ducts in the dermal papilla, and superficial mesh network [60]. The abovementioned imaging of the skin microstructure is useful for studying cellular or microvascular damage during MNA penetration, and the clearer imaging of skin microstructure is also the basis for MNA safety assessment. CLSM can also be combined with the Z-stack scanning to measure the actual depth of the micropores created by the MNA penetration in the skin [61]. The strongest barrier of CLSM compared to OCT is that the penetration depth is only a few hundred microns and is limited to the superficial dermal papillary region [62], because the multiple scattering at deeper depths causes the signal-to-noise ratio to decrease with increasing imaging depth. Due to the limitation of imaging depth, CLSM is not suitable for evaluating the penetration depth of long-sized microneedles.

The common types of depth detection used to evaluate MNA insertion depth with CLSM are shown in Table 1. CLSM often locates the MNA penetration depth by means of fluorescent dyes labeling (Figure 5(c)). Through tomographic imaging of the fluorescent substance of different depths, the visibility of the fluorescent materials is used to evaluate the penetration depth of the MNAs [63], but the intensity of the fluorescent signal is limited within a certain time range [64]. The penetration and diffusion of fluorescent dyes will also make errors in the evaluation of MNA penetration depth, and the final result is often overestimated than that of tissue section [65] or the actual insertion depth. More accurate results are often obtained depending on the inherent fluorescent properties of the skin tissue or loaded active ingredient itself compared to exogenous fluorescent labeling.

### 3.4. Optical Coherence Tomography Imaging

As a nondestructive “optical biopsy” form of imaging technology, OCT allows real-time and in situ imaging of tissue structures in vivo without the need to pretreat or damage the tissue [69], which has great potential for skin imaging applications. The stratum corneum, epidermis, the upper dermis of the skin, skin appendages, and blood vessels show different optical scattering coefficients; thus, the skin can represent a clear hierarchical structure in OCT imaging [70]. This structural information may provide the basis for rapid location and inspection for skin lesions (such as inflammation, edema, infiltration, and necrosis). Conventional OCT used for ophthalmic diagnosis displays strong multilayer tissue scattering and optical inhomogeneities in this challenging human skin imaging process [71]. Therefore, to obtain optimal imaging results, constant adjustment of OCT parameters is essential to maintain the imaging depth and resolution of the skin sample satisfy the requirements.

**Figure 7:**
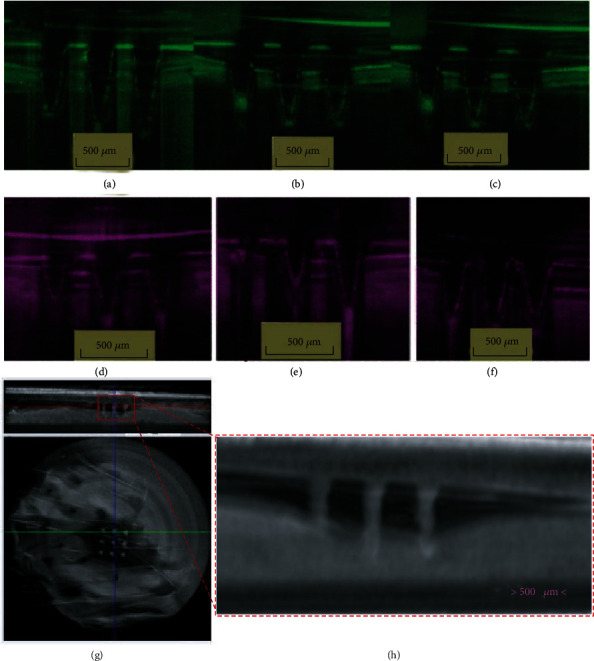
OCT images of MNA insertion. (a–c) MNAs inserted into a porcine corneal (blue) and scleral tissue (purple) prepared from different PVP hydrogels namely (d–f) [72], reprinted with permission from Springer Nature; (g, h) a CT scan image of the MNAs inserted into pig ex vivo skin, showing how to estimate the percentage of the length of the MNAs inserted into the skin [73], reprinted with permission from Elsevier.

Many studies adopted OCT in the research of MNA insertion process, which greatly exerted the strengths of OCT in real-time detection (Figures 7(a)–7(f) and 5(a)).  Table 2 lists some of these studies.

### 3.5. CT Scanning

Computer-assisted tomography (CT) is an imaging technology for clinical diagnosis, which uses collimated X-rays and extremely sensitive detectors for cross-sectional scanning with strong tissue penetration [83]. Micron-scale computed tomography (micro-CT) can provide higher resolution and obtain information about the microstructure and composition of tissues [84]. Loizidou et al. [73] combined micro-CT scan imaging and finite element analysis to study how the geometric composition of the MNAs affects the penetration characteristics. micro-CT uses a series of X-ray scans taken at different angles to generate voxel and visualize MNA insertion in three-dimension (3D) (Figures 7(g) and 7(h)). It is worth noting that it took three hours to use CT scan imaging, during which the rebound characteristics of the skin may affect the original actual penetration effect. Abramson et al. [76] used micro-CT to evaluate the penetration ability of enteric-coated MNAs and developed a luminal unfolding microneedle injector to consistently deliver insulin-loaded dissolvable MNAs into intestinal tissue, which builds a platform to deliver the macromolecule drugs.

Polymer hollow microneedles are preferable choice for enabling to create a small, wearable and minimally invasive closed-loop system. micro-CT is used to study the lumen in the microneedles in order to detect the presence of any blockage due to either debris or tapering owing to the fabrication method [9]. micro-CT scanning technology can help visualize whether all the needles in the array can pierce and penetrate the skin or observe if there are areas in the patch that cannot penetrate the skin due to mechanical failure. However, this approach is subject to certain limitations of radiation and others. Some of these include the difficulty in distinguishing the exact penetration layer of the skin, which hinders the visualization of MNA insertion and limits the quantitative assessment of penetration depth.

## 4. Characterization of MNAs

**Figure 8:**
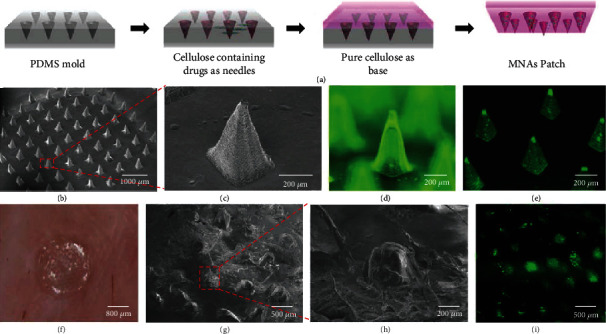
(a) Fabrication process of MNAs. (b) SEM images of LCC-NP-coated MNAs. (c) Magnified SEM images of the MNAs within the red box of (b). (d) Fluorescence microscope image. (e) Confocal image of NBD-PE-labeled NP-coated MNAs. (f) Photograph of MNA patch pressed into porcine skin. (g) SEM images of dissolved MNAs after inserting into skin. (h) Magnified SEM image of MNAs in the red box of (g). (i) Confocal image of porcine skin after treatment with NBD-PE-labeled MNAs [91], reprinted with permission from American Chemical Society (where LCC-NP is lipid-coated cisplatin nanoparticle, NBD-PE represents the 1,2-distearoyl-sn-glycero-3-phosphoethanolamine-N-[amino(polyethylene glycol)-2000] (ammonium salt), a green fluorescent agent).

The physical characteristics of the MNAs affect the insertion effect as well as its in vivo action, which first requires a basic evaluation of certain parameters such as its homogeneity, length, sharpness, stiffness, and perpendicularity [85]. Imaging devices such as stereomicroscope [86], bright-field microscope [87], optical microscope, scanning electron microscope (SEM) (Figures 8(c), 8(g), and 8(h)), and fluorescence stereomicroscope are available for observation.

The distribution of the drug loaded by MNAs includes that of the needle tip, the needle body, and the entire MNA patch. Needle tip loading can ensure precise dosage and complete drug delivery (Figure 3). MNAs are usually labeled with fluorescent dyes such as FITC [29, 88] or rhodamine B [89] and observed by imaging under a fluorescent microscope (Figure 8(d)) or fluorescent Raman spectroscopy [90]. CLSM can effectively evaluate the tomography of microneedle bodies at different angles and characterize the distribution of drugs in the matrix of MNAs by detecting fluorescence signals [91] (Figure 8(e)). High-resolution transmission electron microscopy (HR-TEM) can be used to characterize the nanoparticles loaded in the MNA matrix [65].

## 5. Challenges, Opportunities, and Strategies

The evaluation of MNA skin insertion involves a complex interrelationship and requires finding a match between the microneedle, skin, and imaging techniques. Therefore, it is crucial to select the appropriate imaging modality based on skin characteristics or the composition of MNAs, to improve imaging results by using contrast agents or optical clearing agents (OCAs). Currently, researchers are still in the process of exploring an imaging technique that can allow detection of clear and accurate 3D dynamic imaging in vivo.

### 5.1. Challenges of MNA Imaging In Vivo

**Figure 9:**
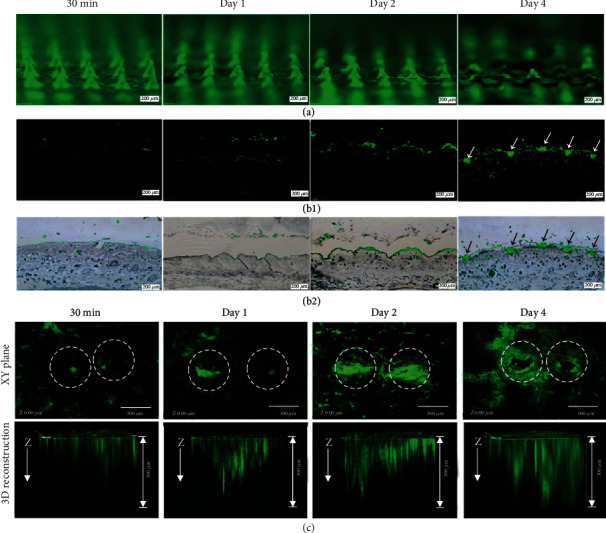
In vivo transdermal delivery of Alexa 488-BSA (green) to the back skin of Sprague−Dawley (SD) rats using P3 MNAs for 30 min, 1, 2, and 4 days [89], reprinted with permission from American Chemical Society. (a) Fluorescence micrographs of Alexa 488-BSA-loaded MNAs after being removed from the skin. Fluorescence images (b1) and merged images of bright-field and fluorescence (b2) of histological sections of skin puncture sites. The arrows in (b1) and (b2) show the fragments of the MNAs left in the skin. (c) Penetration of Alexa 488-BSA (green) within the rat skin pierced by the MNAs for 30 min, 1, 2, and 4 days. Confocal micrographs of the skin surface (upper panel) showing a gradual diffusion of Alexa 488-BSA from the puncture sites (dash circle) to the surrounding skins. 3D confocal reconstruction images of skins (lower panel) showing penetration depth of Alexa 488-BSA in the skin.

The in vivo dissolving and diffusion state is usually observed with the help of bright-field and fluorescence microscope [92] (Figure 9(a) (b1 and b2)), OCT, CLSM (Figure 9(c)), or in vivo optical imaging system (IVIS) in small animals. Also, with the help of near-infrared (NIR), photothermal conversion factors loaded in the drug can be used to determine the location of the drug based on temperature imaging [93].

Labeling the location of drugs, DNA, or proteins with fluorescence or exploiting their inherent fluorescent properties, is common in in vivo imaging techniques, such as in vivo bioluminescence imaging (BLI) and in vivo fluorescence imaging [94]. BLI can be used for directional analysis and simple quantitative calculations but cannot provide information about the depth of the luminescent source within the organism. IVIS imaging system, as a representative of in vivo fluorescence imaging technique, provides accurate localization of tomographic fluorescence imaging and drug distribution [95]. It is worth noting that in the case of mixing fluorescent agent and drug, the observed results only represent the location of the fluorescent agent but not the actual distribution of the drug.

The study of the overall distribution of MNAs after puncture into the body is relatively satisfactory, but its precise kinetic study which involves the inversion calculation is still difficult [95]. 3D structural images can be reconstructed via the difference in scattering coefficient between the dissolvable MNAs' own material and biological tissue, or with the help of fluorescent imaging agents (Figure 5). However, there is still a great gap to achieve accurate quantification and 3D reconstruction imaging technology due to the following challenges:

1. The MNA itself has a limited capacity, with a standard conical needle length of 600 *μ*m and bottom width of 200 *μ*m for an array patch of 10 × 10 needles, whose total volume of needle body is only 0.6 *μ*L, and the imaging agents, drugs, and matrix materials carried are very limited
2. The skin is a complex elastomer that produces constraints and adverse effects during MNA insertion, making the penetration behavior of the MNAs in the arrays nonhomogeneous, i.e., the depth and angle of penetration may not be identical
3. Dissolvable MNAs will gradually dissolve, swell, or degrade after being pierced into the body. The differences between the dissolved, swelled, or degraded states and the solid state, possibly reflected in the differences in the absorption, reflection, refraction, and scattering of light waves
4. It is difficult to maintain the microstructure produced by MNA penetration due to the elasticity of skin tissue and the physical deformation of microneedles, especially when those invasive and destructive observation methods are used
5. In volumetric imaging, there are still certain technical bottlenecks in the resolution and accuracy of the imaging technology

The above issues will greatly increase the complexity of the considerations needed to improve the depth and resolution of the imaging and the 3D reconstruction methods, which may differ from the applications in medical and cell science research.

In addition, the penetration behavior of MNAs is more complex than that of single microneedle. The differences between the artificial simulated skin, animal skin, and human skin will lead to deviations between the predicted value, the actual situation, and the measured results [19]. These are also important issues when determining the penetration depth, drug loading, and distribution of MNAs using imaging techniques. In this regard, noninvasive high-resolution, high-penetration 3D dynamic imaging techniques will provide strong support for the development of MNA technologies and products. Considering the 3R principle (reduction, replacement, and refinement) to be followed in animal experiments, noninvasive imaging technology is of great significance not only for reducing the use of experimental animals, more in line with animal ethics, but also for the precision of detection results.

So far, the research penetration volume of MNAs is still in its initial stage. For example, in skin cosmesis, some studies have evaluated the effect of soluble MNAs loaded with adenosine in reducing wrinkles and quantitatively analyzed the reduction of wrinkles in two groups using a 3D dermatometer [96]. The depth and volume of wrinkles around the eye were measured by the PRIMOS compact based on the digital strip projection technique [24]. These detection methods are of reference significance for the MNA insertion volume, which shows that the demand for obtaining and analyzing the 3D data of skin has markedly increased in recent years.

Similarly, to better evaluate the MNA penetration volume, high-performance and robust algorithms including deep learning methods for MNA image segmentation and extraction are urgently needed. In order to overcome the limitation of multiple low-resolution images decoded from the optical field camera on the accurate 3D surface reconstruction required for tactile palpation, the depth map of generative adversarial networks (GAN) accurately estimates the skin surface depth map with high resolution. This aspect has attracted many research interests. More recently, Hassan et al. [97] presented a residual learning-based framework, dubbed RASP-Net, which adopts computer vision techniques such as atrous spatial pyramid pooling to achieve high-quality segmentation, demonstrating a promising future of computer vision-based MNA penetration volume evaluation.

### 5.2. Opportunities for Imaging Quality Improvement

Every imaging technology is based on the appropriate use of certain optical and other principles. However, the pathway to hardware and principal breakthroughs is not always open and endless. The ability to obtain high-definition images of biological tissue in micro- and nanospaces can also be achieved with the rational use of various imaging enhancers, such as imaging contrast or optical clearing agents, fluorophores, and multifunctional nanocarriers.

#### 5.2.1. Principles of Applicable Imaging Technologies

**Figure 10:**
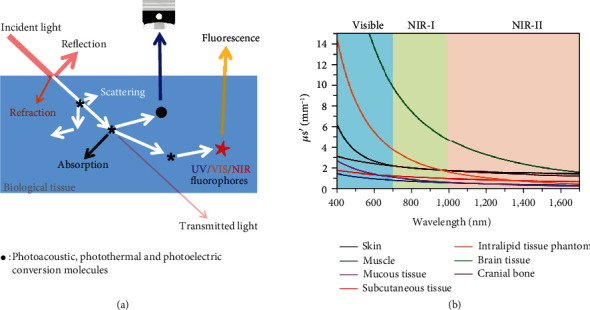
Mechanism for light-based bioimaging. (a) Schematic of light–tissue interactions. (b) Reduced scattering coefficients of different biological tissues and of intralipid scattering tissue phantom as a function of wavelength in the 400-1700 nm region, which covers the visible, NIR-I, and NIR-II windows (blue, green, and red shaded regions, respectively) [100], reprinted with permission from American Chemical Society.

When considering the principles that can guide the choose of appropriate imaging techniques applicable for evaluation of MNA penetration, the differences in the optical principles underlying these imaging techniques need to be clarified. From the perspective of a pharmacologist, photodynamics (PD) and photokinetics (PK) are both important in the optical imaging. The former refers to the action of light on the recipient object (biological tissue or other subjects), such as the effects of photoacoustic, photothermal, photoelectric, and photochemical reactions; the latter refers to the action of biological tissues on light, including reflection, refraction, absorption, diffusion, and scattering (Figure 10(a)).

Biological tissue is a multiphase inhomogeneous media with high scattering of light waves, through which only ballistic, serpentine, and diffuse photons can pass, with ballistic light being very weak and diffuse light being the strongest. The diffuse light is severely scattered in the medium and basically loses the coherence of the incident light, but contains the structural features of the medium, whereby the technique for laminar imaging is called diffuse optical tomography (DOT) with an imaging depth of 1-10 cm and a resolution of 500-1000 *μ*m [53]. In the near-infrared (800-1700 nm) window, where the 650-1100 nm band is also known as the “tissue optical window,” biological tissues have minimal absorption, scattering, and autofluorescence, allowing higher penetration depths for noninvasive or minimally invasive deep tissue imaging [98] (Figure 10(b)). NIR activatable responsive MNAs have been widely used to achieve triggered release of bioactive substances for wound healing, cancer or diabetes treatment, etc. [22, 27, 93, 99].

Conventional fluorescence microscopes (including confocal) cannot achieve high-resolution depth imaging due to strong biological tissue scattering. Therefore, the biggest challenge in optical imaging is to overcome the scattering effect of tissues. OCT operates with ballistic photons, which are scattered only once. Therefore, OCT exhibits a short penetration depth in highly scattering tissue [101]. Confocal microscopic imaging improves the resolution of optical imaging by effectively suppressing the interference of diffracted light and scattered light. Compared with conventional optical microscopy, the light source is replaced by the laser, the scanning unit, and the pinhole on the back focal plane, thereby improving the limited focal depth [102].

TPM possesses the features of both CLSM and two-photon excitation techniques to achieve 3D laminarization through nonlinear optical excitation, allowing nondestructive high-resolution fluorescent molecular imaging and label-free 3D imaging in vivo [103]. This powerful bioanalytical method has the advantages of high photostability, low photodamage, and high spatiotemporal resolution [104]. Unlike CLSM, TPM utilizes a femtosecond, a near-infrared (680-1100 nm) laser, as its standard light source instead of the typical visible laser. In this case, only two-photon absorption can be formed at the focal plane. The outside of the focal plane is not excitable due to low intensity, reducing the light damage to this region. In this sense, TPM is sharper, as it is capable of reaching the submicron resolution when using two-photon excitation fluorescence microscopy (TPEFM) [105, 106].

PAT does not rely on ballistic or backscattered light, unlike OCT, DOT, and fluorescence tomography. As a result, the imaging depth in PAM is relatively large. The scattering of ultrasound signals by biotissues is 2 to 3 orders of magnitude lower than that of optical signals, so using it to reconstruct images can provide deeper imaging depth and higher spatial resolution. On the other hand, PAI indirectly carries out imaging based on the selective absorption of visible light, near-infrared light, or radio frequency electromagnetic waves by different tissues, which is not possible in pure conventional optical imaging. Light absorption by molecules creates a thermally induced pressure jump that launches ultrasonic waves, which are received with acoustic detectors to form images. Additionally, PAM has rich intrinsic and extrinsic optical contrasts and is free of speckle artifacts compared with USI [57].

Regardless of the imaging technology, the working wavelength used for detection is an important factor that affects imaging quality and depth. Whether it is a wide-field microscope or a confocal microscope, the full width at half maximum of the spot (airy disc), which determines the imaging resolution, is proportional to the incident wavelength [107].

#### 5.2.2. Exploration of Imaging Enhancers

In addition to developing new imaging instruments and techniques, designing new imaging agents, such as fluorophores with better quantum yields, photostability, spectral properties, and biocompatibility, is the foremost approach to achieve deep tissue fluorescence imaging.

Undoubtedly, the improvement of efficient fluorophores and the use of multifunctional nanocarriers are important approaches to enhance fluorescence-dependent imaging and expand its applications. Similarly, the continuous development and ingenious use of various imaging contrast agents or optical clearing agents have played a key role in improving the efficacy of various imaging techniques. Therefore, when exploring how to improve the quality and capabilities of imaging techniques, the necessity of using appropriate imaging enhancers must be taken into consideration.

Given the important role of fluorescent contrast agents in TPM and tissue slices, confocal, fluorescence microscope, and live animal imaging, nanomaterials [108] including semiconductor quantum dots, metal nanoclusters, carbon nanomaterials, upconversion nanoparticles, and fluorescent silicon nanoparticles [55, 100, 109, 110], in addition to a variety of functionally different dyes, have been developed and used successively in the last decade. Fluorescent probes with high fluorescence quantum yields and high absorption coefficients are more desirable in the future [111].

Nanoparticles can increase circulation time and imaging brightness relative to single molecule imaging agent, which promotes the rapid development of nanocarriers for NIR imaging with long excitation and emission wavelengths. Additionally, it is important to choose the right fluorescent imaging agent in conjunction with the characteristics of materials used to construct MNAs, especially those fluorescent nanomaterials [109, 110] with increased mechanical properties of MNAs [112, 113] and various microenvironment-responsive fluorescent probes activated by redox, pH, hypoxic, enzyme, viscosity, ATP, and metal ions [111].

Contrast agents are equally important in OCT, USI, TPM, etc. They usually function as OCAs, called tissue optical clearing agents or skin optical clearing agents. Glycerol, glucose, poly(ethylene glycol) (PEG), dimethyl sulfoxide (DMSO), oleic acid, dextran, and some intravenous contrast agents are widely used in biological tissues [114]. PEG-400 can act as an OCA and significantly improved the photoacoustic amplitude for detection of deep-sealed blood vessels, while glycerol alone improved the image quality of shallow vessels. In contrast, DMSO application resulted in decreased photoacoustic amplitude in the in vivo trials [115].

When OCT is used, the depth of light penetration into highly scattered tissues can be improved by OCAs [116]. Studies have shown that glycerol enhances both OCT imaging depth and contrast [117], whereas DMSO only enhances penetration depth [114]. A mixture of fructose [118] or sucrose [119] with PEG-400 and thiazone was used as an OCA, has the optimal capacity of enhancing the OCT imaging performances, decreasing the scattering and the refractive index mismatching, and leads to an improved imaging performance for the deeper tissues. The imaging performance improvement is most likely caused by the OCA-induced dehydration of skin, and the reduction of scattering coefficient (more than ∼40.5%) and refractive index mismatching (more than ∼25.3%) in the superficial (epidermal, dermal, and hypodermal) layers [118].

Combining with OCAs, the imaging performances, including the imaging depth, resolution, contrast, and sensitivity of various optical imaging modalities, e.g., laser speckle contrast imaging, PAM, OCT, TPM, confocal (Raman) microscopy, etc., have been significantly enhanced [118]. Currently, a number of nanomaterials, including liposomes, polymers, micelles, dendrimers, emulsions, quantum dots, and solid nanoparticles have already been used as ultrasound contrast agents (UCA) [120]. In addition, the two-photon absorption properties can readily be improved just by increasing the loading content of aggregation-induced emission (AIE) fluorogen (AIEgen) [104].

### 5.3. Strategies for Appropriate Imaging Modalities

**Figure 11:**
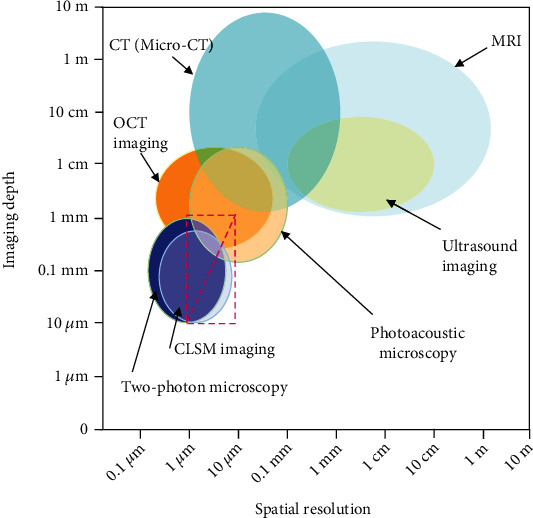
Comparison of the spatial resolution and imaging depth of different imaging techniques used for MNA insertion evaluation. The detection depth and resolution within the dashed box (especially above the diagonal) is the ideal range for in vivo imaging of microneedles.

The imaging analysis of medical products like MNAs, whose sizes are between the macroscopic and mesoscopic scales of matter, is a challenge in terms of the balance and pursuit of resolution and detection depth. Various imaging techniques and devices with different detection depths, resolutions, and sensitivities can achieve different results and are used for different purposes to assess the effect of MNA penetration into the skin or mucosa (Figure 11). The development of OCT, CLSM, TPM, and micro-CT has paved the way for precise imaging-based evaluation of MNA penetration effects. Among them, CT utilizes electromagnetic radiation, which makes it unsuitable for in vivo imaging.

OCT and PAM can image in the millimeter depth range with the axial resolution to the micron level, filling the gap between USI and CLSM. OCT allows real-time imaging and is the most suitable imaging modality for observing the MNA puncture morphology thus far. Meanwhile, PAM is equally competitive in terms of imaging depth, spatial resolution, and speed for MNA evaluation.

Among the mesoscopic diffuse optical imaging techniques, laminar optical tomography (LOT) and spatial frequency domain imaging (SFDI) are well suited for skin characterization since they provide structural and functional information at relatively high spatial resolution (hundreds of microns) and depths of a few millimeters, Therefore, follow-up studies are necessary to elucidate their applications in evaluating MNA penetration quality [121].

**Figure 12:**
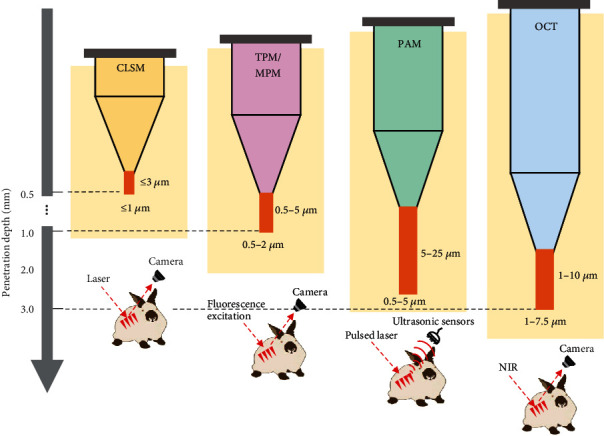
Schematic diagram comparing CLSM, TPM, PAM, and OCT techniques for skin penetration evaluation of MNAs in terms of imaging depth and resolution of lateral and axial (showed in brown squares, aggregated from representative data for the past decade).

In order to obtain a suitable imaging depth and resolution, it is necessary to comprehensively consider the light source parameters (bandwidth and wavelength) and the adaptation of the MNAs [122] (Figures 11 and 12). As shown in Table 2, there are significant differences in imaging depth and resolution among various OCT systems. The imaging depth is dependent on the maximum observable depth of MNA penetration. This depth is determined by the attenuation rates of components in the biological tissues and can be altered through tissue penetrants or refractive index matching fluids.

The resolution determines the clarity of MNA penetration imaging. The lateral resolution of OCT is actually the full width of the spot at the sample, and its best performance can only be achieved at the focal point. Improving the lateral resolution of OCT can be achieved by increasing the numerical aperture of the focusing mirror in the sample arm. However, an increase in lateral resolution means a reduction in the axial field of view, that is, a smaller depth of focus for the Gaussian beam. With the lateral scanning of the OCT, the defocusing phenomenon is more likely to occur. In this case, the sample far away from the zero optical path surface will be severely out of focus, reducing the efficiency of backscattered light collection [123]. The longitudinal resolution of the OCT can be approximated by the coherence length of the light source, which is inversely proportional to the bandwidth. Hence, the axial resolution of the OCT can be improved by increasing the bandwidth of the light source. However, increasing the light source bandwidth means the more serious the effect of dispersion. The main reason is the difference in dispersion characteristics caused by the different medium of the sample and reference arms. The strong dispersion in the system reduces the resolution, which is mainly determined by the bandwidth and the operating wavelength. Therefore, to compensate for this difference, it is necessary to add a dispersion medium to match the sample arm in the reference arm and subtract the additional phase from the postprocessing [124]. The axial resolution of OCT is inversely proportional to the bandwidth of the light source and proportional to the square of the central wavelength of the light source.

From the perspective of detection depth, more and more clinical diagnostic methods have shown considerable advantages. Garcia et al. [125] used Optovue OCT, Visaante OCT and ultrasonic thickness gauge to diagnose opaque cornea, and finally measured depths of 534.03 *μ*m, 523.72 *μ*m, and 529.84 *μ*m, which were consistent with the study of MNA penetration depth. The study used a cornea that is very different from the skin tissue, but it provides an alternative method in terms of the detectable depth.

In OCT measurements of translucent materials, the speckle phenomenon that exists when analyzing depth signal attenuation to gain insight into tissue structure can cause attenuation coefficient images to contain unrealistic fluctuations, making these images less reliable at the voxel level. For this reason, Chang and Bowden [52] estimated the depth-resolved attenuation coefficient from OCT data with speckle, displayed it as an approximately exponential distribution, and finally solved the influence of speckle fluctuation on the depth-resolved recovery of OCT attenuation coefficient. Meanwhile, digital simulation techniques have been used by many researchers to overcome the limitations of OCT resolution, but their applicability is limited by the assumptions of the underlying deep analysis reconstruction technique.

Additionally, the confocal microscope, which is commonly used for skin diagnosis, has a very high resolution (about 1 *μ*m) and a low penetration depth of about 250 *μ*m. It can be connected to OCT clinically to form a line-field confocal optical coherence tomography (LC-OCT) [126], which has better cell resolution than OCT and higher penetration depth than CLSM. LC-OCT can be used to observe the skin surface to the deep dermis (approximately 500 *μ*m) with a high spatial resolution (~1 *μ*m), which is higher than the 5-7.5 *μ*m resolution of ordinary OCT. Also, PAI, high-frequency ultrasound (HFUS), fluorescence Raman spectroscopy imaging, fluorescence lifetime microscopy (FLIM), Odyssey M multimode imaging analysis system, and other imaging technologies have shown increasing potential in the field of MNA penetration research.

Some technologies have been proposed to improve the axial resolution of TPM. Combined with the 4Pi technology, TPM can achieve submicron axial resolution but is restricted to transparent and thin biological samples, which makes it almost impossible to measure the structural and functional information from turbid samples in vivo. If combined with stimulated emission depletion (STED), TPM can break the diffraction limit in both the lateral and axial directions, and the spatial resolution is improved compared with the conventional TPM. Ye et al. [127] proposed a multiframe reconstruction two-photon microscopy (MR-TPM) with adaptive optics correction. This approach enables an almost threefold enhancement of the axial resolution relative to the conventional TPM. Gao et al. [128] developed a novel adaptive optics method for improving the quality of TPM. The method improved the lateral resolution from 1.27 *μ*m to 0.75 *μ*m and the axial resolution from 4 *μ*m to 2 *μ*m with more than a 5-fold increase in fluorescence signal intensity when imaging deep samples (1 mm). These studies altogether show the attractive prospect of TPM technology update.

When dual-band line-field CLSM is used, an imaging depth of ~700-800 *μ*m can be achieved in the skin tissue [129]. The maximum depth of penetration of CLSM is also limited and is reported to be only 1.3 mm. Some scientists [130] developed a novel NIR-II mesoscopic system, which achieves the field of view (FOV) of 7.5 × 7.5 mm^2^, the lateral resolution of 6.3 *μ*m, and the imaging depth up to 2.5 mm. With this system, single capillaries are clearly visible and are available to image 3D at large depths.

With its rich optical absorption contrast and high ultrasound scalability, PAT offers a comprehensive toolbox for life sciences that complements other imaging methods in terms of its contrast mechanism, spatiotemporal resolution, and penetration power. The strong acoustic attenuation and distortion of some biological tissues are a challenge for deep PAT [131], while the decrease in SNR (signal/noise ratio) in deep layers due to light and acoustic attenuation is another concern [132]. To solve this problem, a potential solution is to combine PAT with X-ray CT or other suitable modalities to obtain an accurate 3D model for acoustic correction. Just recently, Zhang et al. [133] developed a reflection-mode submicron-resolution PAM system. By imaging nanospheres and a resolution test chart, the lateral resolution was measured to be ∼0.5 *μ*m with an optical wavelength of 532 nm and an optical numerical aperture of 0.63. The axial resolution was measured at 15 *μ*m.

Although various imaging technologies have been continuously improved to meet the application needs, it is clear that any single method may have its limitations and cannot cope with all the complex demands of multiple objects and scenes. It has become an important trend to use multiple imaging technologies in a smart combination or multimodal imaging, so that the advantages of each complement each other and produce a synergistic optimization effect, in order to obtain the ideal sensitivity, spatial resolution, and imaging speed.

## 6. Conclusion and Outlook

MNAs are being investigated and applied in numerous fields as a convenient, rapid, and novel transdermal drug delivery system, but there is still no acceptable set of regulatory standards [16, 18, 19, 134]. It is necessary and urgent to establish a series of normative criteria and specifications for strict control of the effectiveness and quality of developed MNAs. The approval of the regulatory authorities of pharmaceutical preparations and medical devices is crucial for the safe and effective use of MNAs. Prior to obtaining the product marketing license, a systematic, scientific, and complete product quality control system must be established in accordance with GMP regulations. The imaging science based on the background of a variety of cutting-edge technologies, with the advantages of higher resolution and deeper imaging depth, helps to objectively evaluate the penetration behavior of MNAs both ex vivo and in vivo, thereby developing a comprehensive characterization and evaluation system.

On the other hand, the shift of MNA manufacturing from laboratory assembly to industrial mass production will generate related monitoring and analysis technologies, including imaging technologies, required for MNA manufacturing and quality control in terms of accessibility, operability, reliability, and applicability to multiple scenarios. Taking OCT technology as an example, the main trends are currently focused on large-range imaging, high-speed imaging, etc., which partially meet the needs. Large-range imaging means that the depth of MNA penetration can be probed more precisely, and high-speed imaging makes it possible that real-time video imaging analysis of the process of MNA penetration and the change of the pore channel after extraction. Currently, OCT large-range imaging is mainly used to increase the axial depth by suppressing the conjugate mirror [135, 136] and increasing the spectrometer resolution [137, 138], while OCT high-speed imaging techniques can be briefly grouped into two categories, one relying mainly on increasing the line scan frequency of the line array camera or introducing a high-speed scanning light source and the other by modifying the imaging strategy including setting up multiple line array cameras in the spectrometer and acquiring spectra alternately to achieve an exponential increase in imaging speed [19, 139, 140].

Regardless of the skin models used, either animal skin that similar to human skin or artificial skin manufactured based on real skin parameters [19], the relationship between the imaging mode and the calculation model is often involved in the study of MNAs. Noninvasive imaging methods for real skin punctured by MNAs, tomographic reconstruction techniques like OCT or CT, are currently only in the depth measurement after three-dimensional reconstruction or observation of the overall penetration state. In this sense, the acquisition of insertion depth data is only the analysis and characterization of two-dimensional images. There is a certain overlap between the dermatology imaging technology used in clinical medicine and the imaging technology used for MNA penetration effect, such as OCT, CLSM, TPM, PAT, and USI, so that the development and progress of these technologies are of great significance to the depth or volume of MNA insertion. Achieving precise distinction of subtle differences in the measured subject is not only a matter of imaging technology itself but also a goal to be pursued in precision medicine research at the cellular and human levels.

By accurately analyzing the volume of the MNAs inserted into the body, it provides a more accurate reflection of the total amount of drug in the MNAs delivered into the body than merely observing the insertion length. The exploration of 3D imaging of MNA penetration is still under investigation, but the quantitative analysis methods for MNA loading drugs into the body including high-performance liquid chromatography (HPLC), liquid chromatography-mass spectrometry (LC-MS), gas chromatography-mass spectrometry (GC-MS), and other chromatographic techniques are now widely available and can be easily accessed for utilization. The evaluation of the penetration amount and penetration depth is of great importance for the mechanical properties of the prepared MNAs, drug loading, and other issues. In addition, precision medicine requires higher resolution for fluorescence-based or isotope-labeled imaging techniques to make cellular or subcellular level judgments to study the in vivo distribution of drugs in MNAs [3, 4, 11, 141]. All in all, the characterization and clinical application of MNAs need a multidimensional study. The drug capacity, diffusion rate, penetration efficiency, and fracture force of MNAs are all difficult to characterize only with imaging techniques. It will be an inevitable trend to combine technologies from many fields, including imaging, to evaluate the quality and in vivo pharmacokinetics of MNAs. Simultaneously, the field of imaging technology can also benefit from this unique application, giving rise to new methods or technical improvements.

## Figures and Tables

**Table 1:** The insertion depth of MNAs investigated by CLSM.

Category	Methods	Insertion depth observed	Dimensions of MNAs	Fabricating materials of MNAs	Therapeutic use
Fluorescent labeled	Added FITC fluorescent substance to observe the fluorescence intensity at different depths after MNA penetration [65]	About 650 *μ*m	Shape: cone Height: 900 *μ*m Arrays: 8 × 10	GelCS	Diabetes treatment
	Used rhodamine B as a model drug and its fluorescence characteristics to image different depths of micropores formed by MNAs [63]	About 500 *μ*m	Shape: pyramid Height: 650 *μ*m Arrays: 10 × 10	Alg-APBA/HA	Diabetes treatment
	Used Rhodamine B and Coumarin C6 as model drugs and their fluorescence properties to image MNA penetration depth [66]	About 300 *μ*m	Shape: pyramid Height: 600 *μ*m Arrays: 12 × 12	HA; PVPK17	Psoriasis and arthritis treatment
	Added methylene blue to observe the fluorescence intensity at different depths after MNA penetration [67]	About 125 *μ*m	Shape: pyramid Height: 400 *μ*m Arrays: 10 × 10	HA	Antiaging
Drug labeled	Used the fluorescence characteristics of doxorubicin to study the depth of the micropores after MNA insertion [61]	About 121.5 *μ*m	Shape: pyramid Height: 451.02 ± 8.07 *μ*m Arrays: 10 × 10	PVA	Cancer treatment
Slice reconstruction	Scanned and reconstructed of skin slices punctured by MNAs [68]	/	Shape: pyramid Height: 415 ± 2.6 *μ*m Arrays: 10 × 10	PLGA; PVA	Sustained drug release
Combination of fluorescent labeling and reconstruction	Scanned stereo imaging of the in vitro skin of the penetration distribution of different amounts of drugs labeled with fluorescent dyes [46]	About 155-209 *μ*m	Shape: pyramid Height: 300 *μ*m Arrays: 4 × 4	HA	Intradermal protein delivery
Reduced fluorescence diffusion	After MNA pretreatment, calcein was processed, and the different depths of the micropores of the isolated skin were scanned and imaged [33]	About 115.5 *μ*m	Shape: pyramid Height: 437 ± 14.2 *μ*m Arrays: 10 × 10	PLGA	Immunological diseases

/ : not available. GelCS: gelatin/calcium sulfate hemihydrate composites. Alg-APBA: 3-aminophenylboronic acid-modified alginate.

**Table 2:** OCT-based studies for MNAs insertion depth.

Bio-tissue	Resolution of the instrument	Detection limit or measured depth/range	Dimensions of MNAs	Fabricating materials of MNAs	Therapeutic use
Ocular tissues	Horizontal resolution: 7.5 *μ*m Vertical resolution: 10 *μ*m	Imaged insertion depth is about 600 *μ*m	Shape: cone Height: 800 *μ*m Arrays: 3 × 3	PVP [72]	Model drug delivery (a fluorescent agent)
Skin models; isolated pig skin; abdominal skin of mice in vivo	Horizontal resolution: 15 *μ*m Vertical resolution: 8 *μ*m	Detection depth up to 3.4 mm	Shape: pyramid Height: 2 mm, 800 *μ*m, and 500 *μ*m Arrays: 4 × 4	Silk fibroin [74]	/
Newborn pig skin	Horizontal resolution: 7.5 *μ*m Vertical resolution: 10 *μ*m	Scan width up to 2 mm	Shape: seven different shapes Height: 280-900 *μ*m	PMVE/MA [75]	Antiasthma
Porcine small intestine tissue	Scan resolution up to 1.3 *μ*m	/	Shape: pyramid Height: 600-1200 *μ*m Arrays: 14 × 14	PVP [76]	Diabetes treatment
Newborn pig skin	/	Imaged insertion depth was about 250-300 *μ*m	Shape: cone Height: 600 *μ*m Arrays: 14 × 14	PVP; PVA [77]	Vitamin D3 delivery
Mouse back skin	Horizontal resolution: 10 *μ*m Vertical resolution: ≥5 *μ*m	Imaged insertion depth was about 700 *μ*m	Shape: pyramid Height: 800 *μ*m Arrays: 12 × 12	HA; PVP K90 [78]	Melanoma therapy
Porcine scleral tissue	Horizontal resolution: 7.5 *μ*m Vertical resolution: 10 *μ*m	Imaged insertion depth was about 569.36 ± 15.3 *μ*m	Shape: cone Height: 750 *μ*m Arrays: 3 × 3	PVP; PVA [29]	Model drug delivery (ovalbumin)
Newborn pig ear skin	Horizontal resolution: <7.5 *μ*m Vertical resolution: <5 *μ*m	Detection depth up to 1 mm	Shape: pyramid Height: 650 *μ*m Arrays: 10 × 10	HA; PVP; Maltose [79]	Atrophic scars or photo-aged skin treatment
Full-thickness rat skin	/	Imaged insertion depth was about 300 *μ*m	Shape: pyramid Height: 600 *μ*m Arrays: 12 × 12	HA; PVP [66]	Psoriasis and arthritis treatment
Porcine skin	Horizontal resolution: 10 *μ*m Vertical resolution: 7 *μ*m	The physical scanning range was 2 × 2 × 3 mm^3^	Shape: pyramid Height: about 600 *μ*m	Gelatin; CMC [80]	Diabetes treatment
Neonatal porcine skin	Horizontal resolution: 7.5 *μ*m Vertical resolution: 10 *μ*m	/	Shape: pyramid Height: 630 *μ*m Arrays: 19 × 19	PMVE/MA; PEG 10,000 [81]	/
Mouse skin	Horizontal resolution: 7 *μ*m Vertical resolution: 6 *μ*m	The physical scanning range was 3 × 3 × 2 mm^3^	Shape: pyramid Height: 630 *μ*m Arrays: 351	PVP; PVA [82]	Model drug delivery (rhodamine)

/ : not available. PMVE/MA: copolymer of methylvinylether and maleic anhydride.
